# Phase I/II study of sagopilone (ZK-EPO) plus carboplatin in women with recurrent platinum-sensitive ovarian cancer

**DOI:** 10.1038/bjc.2011.499

**Published:** 2011-11-22

**Authors:** S McMeekin, R Patel, C Verschraegen, P Celano, J Burke, S Plaxe, P Ghatage, M Giurescu, C Stredder, Y Wang, T Schmelter

**Affiliations:** 1 University of Oklahoma Health Sciences Center, Department of Obstetrics and Gynecology, 825 NE 10th Street, Oklahoma City, OK 73104, USA; 2 Comprehensive Blood and Cancer Center, 6501 Truxtun Avenue, Bakersfield, CA 93309, USA; 3 University of Vermont, Hematology/Oncology Unit, Given E-214 – UVM363, 89 Beaumont Avenue, Burlington, VT 05405, USA; 4 Greater Baltimore Medical Center, 6569 North Charles Street, Suite 205, Baltimore, MD 21204, USA; 5 Mercer University School of Medicine (Savannah), 4700 Waters Avenue, Savannah, GA 31403, USA; 6 Rebecca and John Moores UCSD Cancer Center, 3855 Health Sciences Drive # 0987, La Jolla, CA 92093-0987, USA; 7 Tom Baker Cancer Centre, 1331 29 Street NW, Calgary, Alberta T2N 4N2, Canada; 8 Bayer HealthCare Pharmaceuticals, Müllerstrasse 178, Berlin 13353, Germany; 9 Bayer HealthCare Pharmaceuticals, c/o The Courtyard, 30 Worthing Road, Horsham, West Sussex RH12 1SL, UK; 10 Astellas Pharma Global Development, Three Parkway North, Deerfield, IL 60015-2548, USA

**Keywords:** sagopilone, carboplatin, ovarian cancer, epothilones

## Abstract

**Background:**

Sagopilone is the first fully synthetic epothilone in clinical development and has demonstrated promising preclinical activity. This phase I/II, prospective, open-label trial investigated the efficacy and safety of sagopilone plus carboplatin in patients with recurrent platinum-sensitive ovarian cancer (OC).

**Methods:**

In phase I (dose-escalation stage), patients with OC recurring at least 6 months after platinum-containing chemotherapy received 3-h infusions of sagopilone (initial dose of 12 mg m^−2^) followed by carboplatin every 3 weeks, for 2–6 treatment courses. Patients enrolled in phase II received 3-h infusions of 16 mg m^−2^ sagopilone. Efficacy was assessed using modified Response Evaluation Criteria in Solid Tumors (modRECIST) and Gynecologic Cancer InterGroup CA125 criteria. The safety and tolerability of sagopilone were also evaluated.

**Results:**

In all, 45 patients received sagopilone at 12 mg m^−2^ or 16 mg m^−2^. There were 29 confirmed tumour responses (21 modRECIST and 8 CA125) across both treatment groups, indicating that the primary objective of the study was reached. The main adverse events (AEs) reported were peripheral neuropathy (75.6%), fatigue (71.1%) and nausea (64.4%). Grade ⩾3 AEs occurred in 35 patients (77.8%). No deaths related to the study drug were reported.

**Conclusion:**

Sagopilone in combination with carboplatin was effective and toxicities were manageable in patients with recurrent platinum-sensitive OC.

Ovarian cancer (OC) is the leading cause of death in women with gynaecological malignancies in the Western world (Cantù *et al*, 2002). Epithelial OC accounts for nearly 90% of OC (Morrison *et al*, 2007). The incidence of OC increases with age and is most prevalent in the eighth decade of life, with an incidence rate of 57/100 000 (National Comprehensive Cancer Network, 2011).

Early-stage OC (stage I) can often be cured by surgery alone. However, the prognosis for women presenting with an advanced stage (III or IV) is generally poor. Approximately 70% of patients present with advanced-stage OC (Rochet *et al*, 2007). Initial treatment for advanced-stage OC involves surgery (debulking) to remove the majority of macroscopic disease followed by platinum-based chemotherapy (Bristow *et al*, 2002; Galligioni *et al*, 2006; Ozols, 2006; Pignata *et al*, 2006; Morrison *et al*, 2007; National Comprehensive Cancer Network, 2011). Combination chemotherapy with a taxane such as paclitaxel or docetaxel has demonstrated increased response rates and improvements in progression-free survival (PFS) (McGuire *et al*, 1996; Piccart *et al*, 2000; Parmar *et al*, 2003).

Despite the initial high response rates associated with platinum-based therapies, a significant proportion of patients relapse and require second-line therapy. As the majority of these patients are incurable, the ultimate goal of second-line therapy is to improve their quality and length of life (Ushijima, 2010). Women who relapse ⩾6 months after initial therapy are considered to have platinum-sensitive disease (National Comprehensive Cancer Network, 2011). The standard of care for platinum-sensitive recurrent disease usually includes combination therapy with a platinum-based compound (carboplatin) and paclitaxel, gemcitabine or liposomal doxorubicin. Platinum-based chemotherapy with paclitaxel has demonstrated improved response rates and survival when compared with conventional platinum-based monotherapy, as shown by the ICON4 trial (Parmar *et al*, 2003). In a study of carboplatin plus gemcitabine *vs* carboplatin alone for patients with recurrent platinum-sensitive OC, the combined treatment was found to improve PFS (8.6 *vs* 5.8 months; *P*=0.0032) (Pfisterer *et al*, 2005). The response rate was also improved in the combination group (47.2% *vs* 30.9% *P*=0.0016). In the CALYPSO study, combination therapy with carboplatin and liposomal doxorubicin was compared with a regimen of carboplatin and paclitaxel (Pujade-Lauraine *et al*, 2010). Median PFS was improved in the liposomal doxorubicin group compared with the paclitaxel group (11.3 *vs* 9.4 months; *P*=0.005). Patients who respond to primary treatment but relapse within 6 months are considered to have recurrent platinum-resistant OC. Treatment for these patients includes topotecan, gemcitabine and liposomal doxorubicin, with response rates ranging from 6 to 15% however, lengthy remissions are infrequent (Pignata *et al*, 2006; Rose *et al*, 2008; Matsuo *et al*, 2010). There has been increasing evidence to support dose-dense therapy (e.g., extended weekly carboplatin and paclitaxel) for patients with platinum-resistant OC.

Tubulin has been established as a clinically validated target in oncology, and taxanes such as docetaxel and paclitaxel stabilise microtubules and demonstrate significant clinical activity. However, their clinical use is limited by the development of resistance (Fojo and Menefee, 2007). The epothilones are a new class of natural anti-microtubule agents that have a similar mode of action to the taxanes, with potential activity in an expanded spectrum of tumour indications (Cheng *et al*, 2008; Klar *et al*, 2008). Sagopilone (ZK-EPO; Bayer HealthCare Pharmaceuticals, Berlin, Germany) is the first fully synthetic epothilone developed for the treatment of solid tumours that has demonstrated antitumour activity in preclinical models (Klar *et al*, 2006, 2008; Hammer *et al*, 2007b). This compound exhibits greater levels of efficacy than taxanes, has a fast and efficient cellular uptake with no recognition by efflux mechanisms, and has an improved therapeutic window (Klar *et al*, 2006, 2008; Hammer *et al*, 2007a, 2007b; Hoffmann *et al*, 2008). In phase I studies, sagopilone was well tolerated, with peripheral neuropathy being the most common adverse event (AE) (Arnold *et al*, 2009; Schmid *et al*, 2010). Signals of activity were reported in patients with breast cancer, cholangiocellular carcinoma, renal cell carcinoma and other cancer types (Arnold *et al*, 2009; Schmid *et al*, 2010). In patients with platinum-resistant OC, sagopilone has demonstrated activity as a single agent, achieving a confirmed response rate of 16% (Rustin *et al*, 2011). As sagopilone is administered as a Cremophor-free infusion, pre-medication is not required upon administration of this drug (Arnold *et al*, 2009).

The recommended dose of sagopilone for phase II studies is 16 mg m^−2^, with a maximum tolerated dose of 22 mg m^−2^ (Schmid *et al*, 2010). The current paper reports data from a phase I/II trial where sagopilone was administered in combination with carboplatin in women with recurrent platinum-sensitive OC.

## Materials and methods

**Figure 1:**
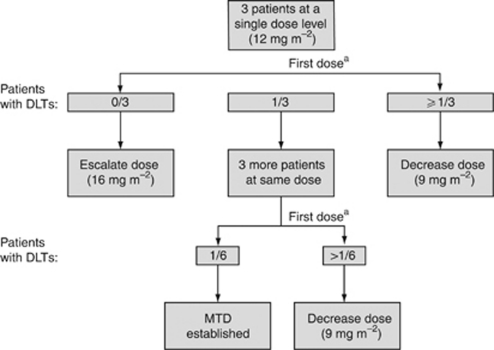
Dose-escalation flow chart. Abbreviations: DTL, dose-limiting toxicity; MTD, maximum tolerated dose. ^a^Patients were allowed to receive additional courses at the dose level at which they began treatment.

This uncontrolled, open-label, multicentre, phase I/II study (ClinicalTrials. gov identifier: NCT00325351) was conducted at 16 trial sites across Canada and the USA. In phase I of the study, the primary objective was to establish the dose of sagopilone, in combination with carboplatin, to be administered to eligible patients (Figure 1). In phase II of the study, the primary objective aimed to determine the efficacy of sagopilone in combination with carboplatin in women with recurrent platinum-sensitive OC. Secondary objectives were to assess the safety and tolerability of sagopilone in combination with carboplatin in these patients.

In phase I, patients received an initial dose of 12 mg m^−2^ sagopilone as a 3-h intravenous infusion, followed by a carboplatin infusion (area under the curve 5, calculated using the Calvert formula) every 21 days. Depending on the safety and tolerability observed after the initial sagopilone dose, the next dose level was planned to be either 9 or 16 mg m^−2^.

All patients involved in the study were to receive 2–6 treatment courses of the study drug. Individuals who were observed to benefit from the treatment received additional courses of sagopilone in combination with carboplatin at the dose at which they started treatment.

The phase II part of the study followed a Simon's two-stage design (Simon, 1989): 15 patients were initially treated (step 1), and if there were ⩾6 responders in these first 15 patients, a further 17 patients were recruited (step 2). Sagopilone was considered effective if ⩾13 patients of the first 32 evaluable patients responded to the treatment, concluding that the true probability of response was >30%. Results data from evaluable patients who had received 16 mg m^−2^ sagopilone in phase I of the study were used for the evaluation of phase II, who were considered as the first patients for step 1.

The study included females aged ⩾18 years with histologically proven epithelial OC, peritoneal cavity cancer or fallopian tube cancer. All patients had disease progression or recurrence following a progression-free and platinum-free interval of at least 6 months after completing first-line platinum-based chemotherapy. All patients had a World Health Organization performance status of 0 or 1 and must not have received prior radiotherapy or immunotherapy within 4 weeks of enrollment or prior chemotherapy within 24 weeks of enrollment. All patients must have had adequate recovery (excluding alopecia) from previous surgeries, radiation and chemotherapy, and have adequate function of major organs and systems. Exclusion criteria included patients who had received prior treatment with epothilones, >1 previous platinum-based line of chemotherapy, previous radiation to the whole pelvis and symptomatic brain metastases requiring whole-brain radiation.

The study followed the Declaration of Helsinki and conformed to Good Clinical Practice guidelines. The protocol was approved by independent ethics committees for each study centre. Written, informed consent was obtained from each patient before entry into the study.

### Assessments

The tumour response in patients with measurable disease was assessed by the maximum reduction in target lesions within six cycles of treatment, using a modified version of the Response Evaluation Criteria in Solid Tumors (modRECIST) (modified from Therasse *et al* (2000) to include an additional category termed ‘unknown’, this being the outcome evaluation when response in either the target or the non-target lesion is unknown and no new lesions have been documented). Responders were defined by a >30% reduction in the size of target lesion compared with baseline. All responses were evaluated by either magnetic resonance imaging or computed tomography. In individuals without measurable disease, tumour response was evaluated using CA125 levels according to Gynecologic Cancer InterGroup criteria (Rustin *et al*, 2004). The maximum reduction in CA125 levels was evaluated within 6 days of treatment and responders were defined by a ⩾50% reduction in CA125 levels compared with the pretreatment sample. The incidence, nature and severity of AEs and serious AEs (SAEs) were graded using the Common Terminology Criteria for Adverse Events version 3.0 (Trotti *et al*, 2003). Standard laboratory assessments such as serum chemistry, blood counts and urinalysis were performed locally.

The primary efficacy variable was the number of patients who responded to treatment. Tumour response was established as best overall response, comprising either a partial response (PR) or a complete response (CR) according to modRECIST. In individuals without measurable disease, response was defined as a confirmed ⩾50% reduction in CA125 levels compared with baseline levels at the start of the treatment. The secondary end points of the study were as follows: duration of response, defined as the time from initial response (where confirmed CR or PR is established) to the time of disease progression; time to disease progression, the time from date of enrollment to study treatment phase to the establishment of objective tumour progression or death from tumour; PFS, the time from the start of treatment to either tumour progression or death; and overall survival, the time from the start of the study until death.

### Statistical analysis

All baseline characteristics, safety variables and demographics were summarised by descriptive statistics and/or frequency tables where appropriate. Efficacy and safety data were analysed from the full analysis set, which included all patients assigned to the study treatment. All time to event variables (i.e., PFS and overall survival) were calculated using Kaplan–Meier estimates and corresponding graphs. The phase II part of the study was planned and evaluated according to a Simon's two-step design to test the null hypothesis that the true probability of response was ⩽30% with a significance level of 10% for the one-sided test, which constituted the primary analysis of the phase II part. With a target response rate of >50%, chosen based on clinical considerations for the target population, the sample sizes led to the power of 80%.

## Results

A total of 45 women (median age 63 years (range 26–78)) enrolled in the study and received at least one treatment course (Table 1). In all, 36 (80.0%) of these patients had measurable disease, and the remaining 9 (20.0%) had non-measurable disease with increased CA125 levels (i.e., twice the upper limit of normal within 3 months and confirmed within 2 weeks before first infusion). The majority of patients (66.7%) were stage IIIC on the International Federation of Gynecology and Obstetrics staging system at first diagnosis, with the most frequent type of cancer being epithelial OC (75.6%). Every patient had undergone initial surgery or biopsy; 21 patients (46.7%) had optimal surgery with no macroscopic residual disease thereafter. All patients had received previous chemotherapy for OC and the majority (95.6%) had received prior taxane therapy. All patients had been pre-treated with a platinum-based chemotherapy; none had undergone radiotherapy.

The first three patients enrolled in the study received 12 mg m^−2^ sagopilone, none of whom experienced a dose-limiting toxicity. The remaining 42 patients were treated at a dose of 16 mg m^−2^, according to dose escalation. These 42 patients completed a total of 206 courses (median of five cycles (range 1–12)), whereas the three patients treated at a dose of 12 mg m^−2^ completed a total of 18 courses (median of six cycles (range 4–8)). Treatment compliance was high, as the mean individual dose of sagopilone during the first six courses was 15.4 mg m^−2^ in the 16 mg m^−2^ group and 12.0 mg m^−2^ in the 12 mg m^−2^ group.

Dose reductions occurred in 11 patients (26.2%) in the 16 mg m^−2^-treatment group only. Twenty-five patients (59.5%) in the 16 mg m^−2^-treatment group and three patients (100%) in the 12 mg m^−2^-treatment group required ⩾1 postponement of the treatment. Dose reductions and postponements were necessitated primarily by the occurrence of AEs. From course 2 onwards, treatment was allowed to be postponed for up to 2 weeks, but only two such postponements were allowed.

The median duration of treatment was five cycles. Overall, 18 patients (40.0%) completed ⩾6 courses of the study medication. Treatment was discontinued prematurely by 27 patients (60.0%): one patient in the 12 mg m^−2^ group and 26 in the 16 mg m^−2^ group. The primary reason for discontinuation was AEs in 23 patients (14 patients experienced neuropathy). Three patients withdrew their consent (one due to AEs) and one patient discontinued the study for other reasons. No deaths related to study medication were reported.

### Efficacy

The primary efficacy end point was analysed using the primary analysis set, which consisted of the first 32 patients enrolled in phase II, all treated with a 16 mg m^−2^ dose of sagopilone. As nine patients from step 1 were responders, the study could continue to step 2, where 12 responders were seen in 17 patients. This resulted in a total of 21 responders (65.6%), which was greater than the 13 responders required to conclude that the true probability of response was >30% and that the study was therefore positive.

**Figure 2:**
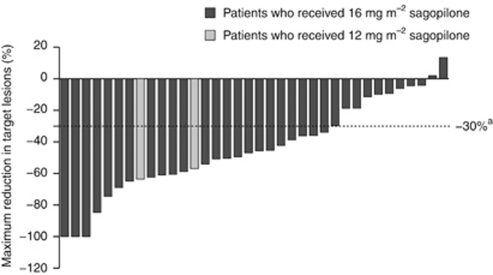
Maximum tumour reduction in target lesions within six cycles of treatment in patients with measurable disease (full analysis set). ^a^Responders were determined by a tumour response rate (or reduction in the size of target lesions) of −30%.

**Figure 3:**
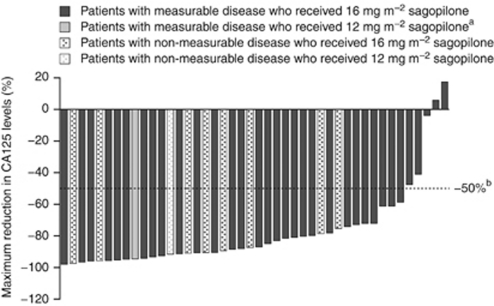
Maximum reduction in CA125 levels within six cycles of treatment (full analysis set). ^a^One patient in the 12mg m^−2^ treatment group was excluded due to unavailable baseline CA125 measurement.^b^ Response was defined as a −50% reduction in CA125 levels compared with the pretreatment sample.

In patients with measurable disease, the maximum reduction in target lesions within six cycles of treatment is shown for each individual in Figure 2. The maximum reduction in CA125 levels for each individual is shown in Figure 3.

Of the 42 patients treated with 16 mg m^−2^ sagopilone, 26 responders (61.9%) were observed (Table 2). In this group, among the 34 patients with measurable disease, three (8.8%) had a CR, 16 (47.1%) had a PR and 11 (32.4%) had stable disease. CA125 was the primary efficacy measurement for the remaining patients without measurable disease. Eight patients were evaluated for a CA125 response and seven had a response (87.5%).

In the 12 mg m^−2^ group, all three patients who were treated had a response. Two patients had measurable disease and both had a PR. One patient was evaluable for CA125 assessment and this patient responded.

Due to the low number of events and patients treated in the 12 mg m^−2^ group, time to event variables were analysed only for patients in the 16 mg m^−2^ group. The median duration of response in the 26 responders was 264 days (95% confidence interval 223–585 days). The median time to tumour progression was 307 days (95% confidence interval 235–347 days). As no deaths were reported during the study, PFS data were identical to the time to tumour progression data.

### Safety

All patients were evaluated for safety. All patients reported AEs, most of which had resolved or were resolving at the end of the study. The highest incidence was observed for peripheral neuropathy, reported in 34 patients (75.6%) (Table 3). Other commonly reported AEs included fatigue in 32 patients (71.1%), nausea in 29 patients (64.4%) and neutropaenia in 18 patients (40.0%) (Table 3). All patients reported at least one AE of grade ⩾2, with peripheral neuropathy (57.8%), fatigue (44.4%) and neutropaenia (40.0%) being the most commonly experienced. Overall, grade ⩾3 AEs were reported in 35 patients (77.8%) (Table 4). The most commonly reported grade ⩾3 AEs were neutropaenia (26.7%) and peripheral neuropathy (22.2%) (Table 4). Overall, grade 4 AEs were reported in nine patients (20.0%): four patients (8.9%) experienced neutropaenia, two patients (4.4%) presented with a decreased neutrophil count, and one case (2.2%) each of febrile neutropaenia, thrombocytopaenia and peripheral sensory neuropathy was reported.

A total of 44 patients (97.8%) experienced at least one AE that was found to be related to the study drug. The most commonly reported drug-related AEs included peripheral neuropathy (75.6%), nausea, (62.2%), fatigue (60.0%) and neutropaenia (37.8%).

Nine of the study patients (20.0%) experienced a total of 27 SAEs during the trial, all of whom received 16 mg m^−2^ sagopilone. SAEs occurring in at least two patients included catheter-related infection, anaemia, nausea, vomiting and pyrexia. Drug-related SAEs were experienced by eight patients. All patients who continued with the treatment had recovered by the end of the study.

## Discussion

The study aimed to investigate the efficacy and tolerability of sagopilone in combination with carboplatin in women with recurrent platinum-sensitive OC. The data from the trial showed that the study met its primary end point, with a total of 21 responders (65.5%) in the first 32 patients enrolled in the phase II part of the study.

The efficacy findings from this study compare favourably with those reported from other second-line platinum-based combination therapies in patients with OC. Published phase II studies of carboplatin plus paclitaxel or gemcitabine or pegylated liposomal doxorubicin combination regimens for platinum-sensitive recurrent OC have reported response rates in the range of 46–91% (Rose *et al*, 1998; Power *et al*, 2009; Bafaloukos *et al*, 2010; Ushijima, 2010). A similar range of response rates (47–76%) was reported in phase III studies of platinum-based combination therapies (Parmar *et al*, 2003; Pfisterer *et al*, 2005; Pujade-Lauraine *et al*, 2010; Ushijima, 2010). The response rate in the present study was 64.4% however, the population was predominantly patients with modRECIST measurable disease, suggesting that the high response rate is valid. In a single-arm study evaluating a platinum combination therapy in patients with platinum-sensitive recurrent OC, one must be cautious about estimating the effect of the addition of a novel agent, in this case sagopilone, to platinum. In an effort to confirm any significant activity, the threshold established in this study for describing the combination as clinically interesting was 50%. In the Gynecologic Oncology Group's phase II programme for evaluating platinum combination therapies in platinum-sensitive recurrent OC, it was suggested that if carboplatin combinations result in a response rate of ⩽40%, it is of no clinical interest (Secord *et al*, 2008) and, conversely, if the true response is ⩾60%, further study is clearly indicated.

The toxicity profile of sagopilone in combination with carboplatin is broadly comparable with that previously observed in patients treated with paclitaxel/platinum-based treatment, in terms of the most commonly reported AEs. In our study, the most frequently reported AEs, in 40–76% of patients, included neutropaenia, nausea, fatigue and peripheral neuropathy. In the phase III CALYPSO study, anaemia, nausea, sensory neuropathy, neutropaenia, fatigue and alopecia were the most commonly reported AEs in 64–90% of patients receiving paclitaxel plus carboplatin (Pujade-Lauraine *et al*, 2010). In the phase III ICON4/AGO-OVAR-2.2 study, neurological and haematological toxic effects, grades 2–4 nausea and vomiting, and alopecia were experienced by 20–86% of patients receiving paclitaxel plus a platinum-based chemotherapy (Parmar *et al*, 2003). Neutropaenia was the most commonly experienced grade ⩾3 AE (27%) in our study. The incidence of grade 3 or 4 neutropaenia and haematological toxic effects was 46% in the CALYPSO study and 29% in the ICON4/AGO-OVAR-2.2 study, respectively (Parmar *et al*, 2003; Pujade-Lauraine *et al*, 2010).

Neurotoxicity events are one of the most clinically relevant events related to sagopilone (Beer *et al*, 2008; Daud *et al*, 2009; Schmid *et al*, 2010; Rustin *et al*, 2011). In the present study, peripheral neuropathy was the most commonly reported drug-related AE experienced by 75.6% of patients across all grades, the majority of which were reported at grade 1 or 2. Grade ⩾2 peripheral neuropathy was reported by 57.8% of patients in our study. In the ICON4/AGO-OVAR-2.2 study, grades 2–4 neurological toxicities were reported in 20% of patients receiving a combination of paclitaxel and a platinum-based chemotherapy (Parmar *et al*, 2003). Similarly, in the CALYPSO study, 27% of patients receiving paclitaxel plus carboplatin experienced sensory neuropathy of grade ⩾2 (Pujade-Lauraine *et al*, 2010).

Sensory neuropathy is a major complication associated with the use of taxanes and epothilones; therefore, the high proportion of patients reporting sensory neuropathy was not unexpected following treatment with sagopilone, especially in a population pre-treated with taxanes. However, the sensory and motor symptoms of neuropathy can be severely disabling and can have a significant impact on a patient's quality of life (De Grandis, 2007). Strategies which reduce the neuropathy rates, including improved patient selection, dose scheduling modifications and earlier dose reductions, should be pursued. In addition, a number of strategies are being studied to overcome sensory neuropathy, including the use of acetyl-L-carnitine (De Grandis, 2007) and oral glutamine (Wang *et al*, 2004). In a recent phase II study to evaluate the use of acetyl-L-carnitine in the prevention of sagopilone-induced peripheral neuropathy, the incidence of grade 3 or 4 peripheral neuropathy in patients with OC was significantly lower in those receiving acetyl-L-carnitine (20%) compared with those receiving placebo (41%) (*P*=0.04) (Lhomme *et al*, 2011).

This study successfully met the primary end point and demonstrates proof of concept for treatment with sagopilone plus carboplatin in women with recurrent platinum-sensitive OC. This combination therapy was feasible in pre-treated patients, with manageable toxicity and activity worthy of further exploration, although vigilance must be exercised in the prevention and management of neuropathy.

## Figures and Tables

**Table 1:** Patient characteristics

	**Number of patients (%) (*n*=45)**
*Age, median (range), years*	63 (26–78)
	
*FIGO stage at diagnosis*	
IC-IIIB	12 (26.7)
IIIC	30 (66.7)
IV	3 (6.7)
	
*WHO performance status*	
Grade 0	44
Grade 1	1
	
*Type of cancer*	
Fallopian tube	3 (6.7)
Peritoneal cavity	8 (17.8)
Epithelial ovarian	34 (75.6)
	
*Histology findings*	
Serous cells	28 (62.2)
Adenocarcinoma	13 (28.9)
Clear cell type	2 (4.4)
Endometrioid cells	1 (2.2)
Other	1 (2.2)
	
*Histological grade at first diagnosis*	
1	3 (6.7)
2	9 (20.0)
3	24 (53.3)
Unknown	9 (20.0)
	
*Disease status*	
Measurable disease	36 (80.0)
Sum of longest diameters (mm) of target lesions at screening, median (range)	67.5 (19–269)
Non-measurable disease	9 (20.0)
CA125 serum level (U ml^−1^), median (range)	124.9 (17.5–561.0)
CA125 serum level (U ml^−1^) in patients with non–measurable disease, median (range)	167 (77–428)
	
*Prior chemotherapy*	
1	42 (93.3)
2	3 (6.7)
Prior taxane therapy	43 (95.6)
	
*Platinum-free interval, months* (%)	
6–12	11 (24.4)
>12–24	19 (42.2)
>24	15 (33.3)

Abbreviations: FIGO=International Federation of Gynecology and Obstetrics; WHO=World Health Organization.

**Table 2:** Best overall tumour response

		**Full analysis set**	
**Response assessment**	**Response category**	**16 mg m^−2^, *n* (%), *N*=42**	**12 mg m^−2^, *n* (%), *N*=3**
modRECIST		*N*=34	*N*=2
	Response (CR+PR)	19 (55.9)	2 (100)
	CR	3 (8.8)	0
	PR	16 (47.1)	2 (100)
	SD	11 (32.4)	0
	PD	1 (2.9)	0
	ND/UNK/NA	3 (8.8)	0
			
CA125		*N*=8	*N*=1
	Response	7 (87.5)	1 (100)
			
Overall response		*N*=42	*N*=3
	Response	26 (61.9)	3 (100)
	Overall	29/45=64.4%	

Abbreviations: CR=complete response; modRECIST=modified Response Evaluation Criteria in Solid Tumors; ND/UNK/NA=not done/unknown/not available; PD=progressive disease; PR=partial response; SD=stable disease.

**Table 3:** Most frequently (>10% of all patients) reported adverse events (Common Terminology Criteria for Adverse Events, all grades)

	**16 mg m^−2^, *N*=42, *n* (%)**	**12 mg m^−2^, *N*=3, *n* (%)**	**Total**, ***N*=45, *n* (%)**
Any event	42 (100)	3 (100)	45 (100)
Peripheral neuropathy	33 (78.6)	1 (33.3)	34 (75.6)
Fatigue	30 (71.4)	2 (66.7)	32 (71.1)
Nausea	28 (66.7)	1 (33.3)	29 (64.4)
Neutropaenia	16 (38.1)	2 (66.7)	18 (40.0)
Diarrhoea	16 (38.1)	1 (33.3)	17 (37.8)
Arthralgia	14 (33.3)	1 (33.3)	15 (33.3)
Constipation	13 (31.0)	2 (66.7)	15 (33.3)
Anaemia	12 (28.6)	2 (66.7)	14 (31.1)
Anorexia	14 (33.3)	0	14 (31.1)
Headache	12 (28.6)	0	12 (26.7)
Vomiting	12 (28.6)	0	12 (26.7)
Insomnia	11 (26.2)	0	11 (24.4)
Myalgia	10 (23.8)	0	10 (22.2)
Pain in extremity	9 (21.4)	1 (33.3)	9 (20.0)
Urinary tract infection	8 (19.0)	1 (33.3)	9 (20.0)
Dizziness	7 (16.7)	1 (33.3)	8 (17.8)
Muscular weakness	7 (16.7)	0	7 (15.6)
Neutrophil count decreased	7 (16.7)	0	7 (15.6)
Pyrexia	6 (14.3)	1 (33.3)	7 (15.6)
Upper respiratory tract infection	5 (11.9)	1 (33.3)	6 (13.3)
Abdominal pain	5 (11.9)	0	5 (11.1)
Dehydration	5 (11.9)	0	5 (11.1)
Musculoskeletal pain	5 (11.9)	0	5 (11.1)
Pain	5 (11.9)	0	5 (11.1)
Thrombocytopaenia	3 (7.1)	2 (66.7)	5 (11.1)

**Table 4:** Number of patients with most frequently reported adverse events of Common Terminology Criteria for Adverse Events grade ⩾3^a^

	**16 mg m^−2^,** ***N*=42, *n* (%)**	**12 mg m^−2^, *N*=3, *n* (%)**	**Total,** ***N*=45, *n* (%)**
Any event	33 (78.6)	2 (66.7)	35 (77.8)
Neutropaenia	11 (26.2)	1 (33.3)	12 (26.7)
Peripheral neuropathy^b^	10 (23.8)	0	10 (22.2)
Decreased neutrophil count	4 (9.5)	0	4 (8.9)
Hypokalemia	4 (9.5)	0	4 (8.9)
Arthralgia	4 (9.5)	0	4 (8.9)
Anaemia	3 (7.1)	0	3 (6.7)
Drug hypersensitivity	3 (7.1)	0	3 (6.7)
Catheter-related infection	3 (7.1)	0	3 (6.7)
Thrombocytopaenia	0	2 (66.6)	2 (4.4)
Fatigue	2 (4.8)	0	2 (4.4)
Dyspnoea	2 (4.8)	0	2 (4.4)

a Adverse events occurred in at least two (4.4%) patients within the total population.

b Includes balance disorder, cranial neuropathy, hypoaesthesia, neuralgia, neuropathy peripheral, paraesthesia, peripheral motor neuropathy and peripheral sensory neuropathy.

## Appendix

The following investigators and institutions also participated in the trial:

Alexander Burnett, University of Arkansas, Fayetteville, AR, USA; Allan Covens, Toronto Sunnybrook Regional Cancer Centre, Toronto, Ontario, Canada; Ana Maria López, Arizona Cancer Center at UMC North, Tucson, AZ, USA; David Martin, Baptist Hospital, Knoxville, TN, USA; James Mason, Scripps Cancer Center, La Jolla, CA, USA; Michael Method, Northern Indiana Cancer Research Consortium, South Bend, IN, USA; Brigitte Miller, Wake Forest University School of Medicine, Winston-Salem, NC, USA; Carolyn Muller and Teresa Rutledge, University of New Mexico Cancer Center, Albuquerque, NM, USA; Peter Rose, MetroHealth Medical Center, Cleveland, OH, USA; Dennis Schribner, Carilion GYN Oncology Associates, Roanoke, VA, USA.
